# Putting Oneself in the Body of Others: A Pilot Study on the Efficacy of an Embodied Virtual Reality System to Generate Self-Compassion

**DOI:** 10.3389/fpsyg.2019.01521

**Published:** 2019-07-02

**Authors:** Ausiàs Cebolla, Rocío Herrero, Sara Ventura, Marta Miragall, Miguel Bellosta-Batalla, Roberto Llorens, Rosa Ma Baños

**Affiliations:** ^1^Department of Personality, Assessment and Psychological Treatments, University of Valencia, Valencia, Spain; ^2^CIBER of Physiopathology of Obesity and Nutrition (CIBEROBN), Madrid, Spain; ^3^Department of Basic and Clinical Psychology and Psychobiology, Universitat Jaume I, Castelló, Spain; ^4^Neurorehabilitation and Brain Research Group, Instituto de Investigación e Innovación en Bioingeniería, Universitat Politècnica de València, Valencia, Spain; ^5^Servicio de Neurorrehabilitación y Daño Cerebral de los Hospitales Vithas-NISA, Fundación Hospitales NISA, Valencia, Spain

**Keywords:** compassion, virtual reality, body swapping, full body illusion, self-compassion, mindfulness, meditation

## Abstract

Compassion-based interventions (CBIs) have been shown to be effective for increasing empathy and compassion, and reducing stress, anxiety, and depression. CBIs are based on constructive meditations where imagery abilities are essential. One of the major difficulties that participants report during the training is the difficulty related to imagery abilities. Virtual reality (VR) can be a useful tool to overcome this limitation because it can facilitate the construction and sustainment of mental images. The machine to be another (TMTBA) uses multi-sensory stimulation to induce a body swap illusion. This system allows participants to see themselves from a third perspective and have the illusion of touching themselves from outside. The main objective of the present study was to analyze the efficacy of a self-compassion meditation procedure based on the TMTBA system versus the usual meditation procedure (CAU) in increasing positive affect states, mindful self-care, and adherence to the practice, and explore the influence of imagery abilities as moderators of the effects of the condition on adherence. A sample of 16 participants were randomly assigned to two conditions: TMTBA-VR and CAU. All participants had to listen to an audio meditation about self-compassion and answer questionnaires before and after the training. The TMTBA-VR condition also had a body swap experience at the end of the meditation while listening to self-compassionate messages. Afterward, they were invited to practice this meditation for 2 weeks and then measured again. After the compassion practice, both conditions significantly increased positive qualities toward self/others, decreased negative qualities toward self, and increased awareness and attention to mental events and bodily sensations, with no differences between the conditions. After 2 weeks, both conditions showed a similar frequency of meditation practice and increases in specific types of self-care behaviors, with the frequency of clinical self-care behaviors being significantly higher in TMTBA. Finally, lower imagery ability in the visual and cutaneous modality were moderators of the efficacy of the TMTBA (vs. CAU) condition in increasing adherence to the practice. Embodied VR could be an interesting tool to facilitate and increase the efficacy of CBIs by facilitating the construction of positive and powerful mental images.

## Introduction

Compassion-based interventions have been shown to be effective in increasing empathy and compassion (Brito et al., 2018), and reducing stress, anxiety, and depression (Kirby et al., 2017). They have been used in clinical settings, such as oncology (Gonzalez-Hernandez et al., 2018) or personality disorders (Feliu–Soler et al., 2017). Compassion refers to “*the feeling that arises in witnessing another’s suffering and that motivates a subsequent desire to help*” (Goetz et al., 2010, p. 351). When this feeling is focused on oneself, it is called self-compassion, defined as individuals’ ability to respond to their own suffering with warmth and the desire to alleviate their own pain (Neff and Dahm, 2015).

Compassion-based interventions use different techniques and meditations to achieve the objective of increasing self-compassion and compassion skills, such as focused attention meditations to calm the mind and, mainly, the family of constructive meditations (Dahl et al., 2015). In this family of meditation practices, the meditator purposefully strengthens his/her natural capacity for loving kindness and compassion by intentionally generating compassionate thoughts, feelings, and motivations toward different objects, including him/herself (Brito et al., 2018).

In order to induce and train these positive mental states, the family of constructive meditations requires the use of mental imagery abilities. Surprisingly, the impact of these imagery skills on CBIs has not been studied, even though one of the major difficulties that participants report during the training is related to these imagery abilities. According to Pearson et al. (2013), there are four different mental imagery skills related to different processes that could be interacting with these types of meditation: (a) creation, (b) sustainment, (c) inspection, and (d) transformation of mental images. In creation, the meditators have to select the type of images or elements that will be used in the meditation. In the second process, the sustainment of the mental image, research shows that after 250 ms (the time necessary for eye movement) (Kosslyn, 1994), the image starts to decay. Thus, participants usually have to deal with the frustration of not being able to sustain the image long enough, which could interact with their positive emotional state. The third aspect, inspection, refers to the interpretation of an object-based characteristic or spatial property of this generated image. For example, the lack of definition (blurred) and vividness of the mental image is also a significant factor. Finally, transformation includes the capacity for rotation and restructuring.

Although the effects of compassion and self-compassion training are well known, the factors that predict why the training works for some people and not for others have been understudied. In this regard, the absence of adequate training in the ability to create, sustain, inspect, or transform mental images may impede the expected positive effects of compassion training. This lack of ability can lead people to struggle with steps prior to the compassion itself, and this experience can discourage people from continuing the necessary training to develop the compassion skills, like self-care or positive qualities (compassion, equanimity, joy, or loving kindness).

Virtual reality can be a useful tool to overcome this limitation because it can help to construct, sustain, inspect, and transform mental images. VR can be considered an advanced imagery system and an experiential form of imagery that is as effective as reality in inducing cognitive, emotional, and behavioral responses (Day et al., 2004). VR has been used to train compassion and self-compassion. For instance, Slater’s group studied how the use of virtual bodies can promote compassion and self-compassion by analyzing the effects of self-identification with virtual bodies within immersive VR on increasing self-compassion in individuals with high self-criticism and depression (Falconer et al., 2014) showing how could be effective in reducing depression severity and self-criticism. The same group investigated how an embodied black avatar decreases racial prejudice and changes negative interpersonal attitudes (Peck et al., 2013). Bailenson’s group also studied how an embodied avatar in VR can make people more altruist. For example, participants embodied a Superman avatar, and the results showed that they felt more helpful after the experiment (Rosenberg et al., 2013).

All these studies use embodied VR systems, which is a cognitive science approach that emphasizes, among other aspects, the subjective experience of using and “having” a body. This paradigm has been used to generate Full Body Illusions (Ehrsson, 2007) and body swapping experiments, which have become an increasingly popular method for investigating how illusory ownership of an entire fake or virtual body affects various aspects of bodily perception and experience. Thus, VR allows individuals to be present not only in the environment, but also in someone else body. VR allows the person to be “inside” another body (e.g., another person or animal), creating a body swap experience that makes it possible to study the embodiment processes, as well as emotional states such as empathy or compassion (Peck et al., 2013; Rosenberg et al., 2013; Falconer et al., 2014).

The Machine to be another is a low-budget body swapping system designed to address the relationship between identity and empathy through the use of multi-sensory stimulation (visual, cutaneous, proprioceptive, and auditory) to induce a body swap illusion (Oliveira et al., 2016). It allows the user to have an immersive experience of seeing him/herself in the body of another person –a performer– (Bertrand et al., 2018). The TMTBA is connected to the head-mounted display (an Oculus Rift), and the performer’s first-person perspective is captured by a camera controlled by the user’s head movements, showing the torso, legs, and arms of the performer’s body. The user, through the Oculus Rift, sees the image captured by the camera, creating the illusion of being another person, and seeing him/herself from a third-person point of view.

As mentioned above, this system could overcome some limitations of imagery skills, and it can be combined with self-compassion meditations to generate a powerful emotion response of self-compassion and, therefore, increase adherence to the meditation practice and its effects. Thus, the main objectives of this study are to analyze the effects of a self-compassion meditation supported by the TMTBA-VR system, compared to usual practice (only audio) and analyze whether the imagery skills would moderate the effect of the condition on the adherence to meditation practice after 2 weeks. The main hypothesis are divided in two groups, on one hand it is expected effects before and after the meditation supported by TMTBA-VR, showing effectiveness (1) to increase positive qualities toward self/others, decrease negative qualities toward self/others, and increase awareness and attention to the present experience immediately after a compassion practice; and, on the other hand, it is expected an effect after 2 weeks of practice, showing that the participants who received the TMTBA-VR condition will (2) increase adherence to mediation practice, the frequency of self-care behaviors, and positive affect, and decrease negative affect, after 2 weeks compared to usual practice. Furthermore, it is expected that imagery skills will moderate these results.

## Materials and Methods

### Participants

The study was approved by The Ethics Committee at the University of Valencia (Spain), with registration number: H1513592028862. The size of the sample has been determined with the G-Power program, taking as a measure of effect size 0.8 (probability of alpha = 0.05) from the results obtained in the Zeng et al.’s (2015) CBIs meta-analysis of estimating the need to include a sample of 16 participants. The sample was composed of 16 students from the University of Valencia; 75% were female, and the mean age was 30.56 (*SD* = 10.86), ranging from 21 to 59. Participants were allocated to one of two conditions: Guided meditation or Guided meditation supported by TMTBA. The inclusion criteria were: (a) being older than 18 years; and (b) having a good level of Spanish or Valencian. The exclusion criteria were: (a) having a current diagnosis of a psychological disorder; (b) currently undergoing psychological treatment; (c) substance use or abuse; and (d) being a regular practitioner of any meditation practice (more than 5–6 times per week). The screening was completed by 22 participants, but only 16 met the criteria.

### Measures

#### Sociodemographic, Psychological, and Practice-Related Meditation Variables Questionnaire

An *ad hoc* questionnaire was made to collect information about age, gender, highest education level attained, history of mental or chronic illness, use or abuse of drugs, current psychological treatments, and experience with mindfulness and other meditation practices. This questionnaire was administered as part of the screening process for the study.

#### Patient Health Questionnaire-9 (Kroenke et al., 2001; Wulsin et al., 2002)

This is a 9-item questionnaire that measures the presence of depressive disorders, rated on a 0–3 scale. Total scores range from 0 to 27; higher scores indicate higher levels of depressive symptoms. The PHQ-9 has been shown to have good psychometric properties. This questionnaire was administered as part of the screening process for the study. A Spanish adaptation performed by the authors was used and showed adequate internal consistency for the total score (α = 0.66).

#### The Generalized Anxiety Disorder Questionnaire (Spitzer et al., 2006; Ruiz et al., 2011)

This is a one-dimensional self-administered scale used to detect the presence of the symptoms of anxiety. It is an efficient, quickly applied, reliable, and valid instrument, with a scoring scale from 0 to 3 (0 = nothing, 3 = almost every day). The GAD-7 has demonstrated good internal consistency and test-retest reliability, as well as convergent, construction, criterion, procedural, and factorial validity for the GAD diagnosis (Spitzer et al., 2006; Löwe et al., 2008). This questionnaire was administered as part of the screening process for the study, and internal consistency was adequate for the total score (α = 0.82).

#### Betts’ Questionnaire Upon Mental Imagery (Sheehan, 1967; Campos and Pérez-Fabello, 2005)

It consists of 35 items about imagery vividness, rated on a 7-point scale (1 = image perfectly clear and as vivid as the actual experience; 7 = no image present at all, you only “know” that you are thinking of the object). Higher scores indicate low imagery in the seven sensory modalities. Scores on the subscales were calculated according to the Spanish validation (Campos and Pérez-Fabello, 2005), which had adequate internal consistency. This questionnaire was administered in order to use the subscales as moderator variables for the study, and internal consistency was adequate for all the subscales: gustatory and auditory (α = 0.93), kinesthetic (α = 0.98), organic (α = 0.94), visual (α = 0.96), auditory (α = 0.87), and cutaneous (α = 0.92).

#### Positive and Negative Affect Schedule (Watson et al., 1988; Robles and Páez, 2003)

It includes 20 items that evaluate positive affect (10 items) and negative affect (10 items) on a 5-point scale. Respondents indicate how frequently they experience an emotion within a given time framework; in this study, we asked for the past 2 weeks. It has shown good properties of validity, convergence, and divergence, in addition to being a brief, reliable self-report measure. This questionnaire was administered at baseline and at the 2-week follow-up, and internal consistency was adequate for negative PANAS (α = 0.79–0.91) and positive PANAS (α = 0.94–0.95) across the administrations.

#### State Mindfulness Scale (Tanay and Bernstein, 2013)

It has been developed to measure the levels of awareness and attention to present experience during a specific period of time (in the present study, we used the duration of the intervention as a temporal framework) and context (in this case, the self-compassion intervention). The scale is composed of 21 items and includes two subscales: state mindfulness of bodily sensations (6 items) and state mindfulness of mental events (15 items). The scale has shown strong internal consistency reliability for both subscales, as well as the total scale. The scale has also shown and adequate construct validity through positive correlations with a state mindfulness measure and incremental sensitivity to change through demonstrated increases in SMS scores following a mindfulness meditation practice. This questionnaire was administered before and after the compassion intervention. A Spanish adaptation performed by the authors was used and showed adequate internal consistency for the bodily sensations subscale (α = 0.75–0.78) and the mental events subscale (α = 0.93–0.94) across administrations.

#### Self-Other Four Immeasurable Scale (Kraus and Sears, 2009)

It is a measure designed to assess the four main qualities of Buddhist teachings: loving kindness, compassion, joy, and acceptance toward both the self and others. It is a 16-item scale, rated on a five-point Likert scale. The scale has a structure composed of four subscales: positive qualities toward self, positive qualities toward others, negative qualities toward self, and negative qualities toward others. All the subscales have high internal consistency and have shown appropriate construct, convergent, and discriminant validity (Kraus and Sears, 2009). This questionnaire was administered before and after the compassion intervention. A Spanish adaptation performed by the authors was used and showed adequate internal consistency for positive qualities toward self (α = 0.78–0.86), positive qualities toward others (α = 0.70–0.87), and negative qualities toward self (α = 0.83–0.86) across administrations, but not for negative qualities toward others (α = 0.39–0.42).

#### Mindfulness Self-Care (Cook-Cottone and Guyker, 2018)

It is a 33-item scale that measures the self-reported frequency of self-care behaviors in 6 specific domains and on more global practices of self-care. The MSCS asks participants to rate the frequency of self-care behavior in the past 2 weeks. The items are rated on a 5-point Likert scale ranging from 1 (Never or 0 days) to 5 (Regularly or 6 to 7 days). Responses to all items are totaled, with higher scores representing increased frequency of self-care behaviors. The MSCS total and subscales have strong internal consistency reliability. This questionnaire was administered at baseline and at the 2-week follow-up. A Spanish adaptation performed by the authors was used and showed adequate internal consistency for supportive relationships (α = 0.60–0.69), mindful awareness (α = 0.88–0.90), self-compassion and purpose (α = 0.78–0.87), supportive structure (α = 0.59–0.71), and general self-care practices (α = 0.77–0.84) across administrations, but not for physical care (α = 0.45–0.68), mindful relaxation (α = 0.17–0.81), and clinical self-care practices (α = 0.19–0.66).

#### Adherence Question

Adherence to the self-compassion practice was measured at follow-up using a single question. Participants were asked to register the frequency of their meditation practice in the past 2 weeks. Participants rated the frequency on a 5-point Likert scale: 1 = never; 2 = 1–2 times per week; 3 = 3–4 times per week; 4 = 5–6 times per week; 5 = every day.

#### Embodiment in TMTBA Questionnaire

It is an adaptation of the original questionnaire to assess the Rubber Hand Illusion experience developed by Longo et al. (2008), to assess the strength of embodiment elicited by the TMTBA during the study, by asking, for example, about the experience of being located in the performer’s body. The scale is composed of 10 items rated on a Likert-type scale ranging from 1 (strongly disagree) to 7 (strongly agree). The scale contains 3 subscales: 5 items assess body-ownership, 3 items assess location, and the remaining 2 items assess agency.

### Procedure

Students were invited to participate in a study aimed to increase their compassionate skills. Potential participants contacted researchers via email and received access to the screening assessment and the informed consent (T1). Participants who fulfilled the inclusion and exclusion criteria were randomly assigned to one of the two study conditions: usual meditation (CAU) or Meditation through the Machine to be another (TMTBA-VR), using the Random Allocation Software 2.0. Participants came to the laboratory and filled out the pre-assessment (T2). Once they had finished, they performed the meditation. Participants in the CAU condition were seated in a quiet room and listened to a recorded audio with a traditional meditation. Participants in the TMTBA-VR condition followed the steps explained in Section “The TMTBA-VR Condition” below. Both conditions used the same meditation audio. The meditation used was focused in the generation of a self-compassionate state inviting participants to think about themselves while they were kids, at the end a self-compassionate mantras was also used. Once participants had finished the meditation, they completed the post-assessment questionnaires (T3). Participants were instructed to practice the meditation for the following 2 weeks. For this purpose, all participants received an audio track with the meditation performed in the laboratory session. At the end of the 2 weeks, participants completed the follow-up assessment (T4).

### The TMTBA-VR Condition

**FIGURE 1:**
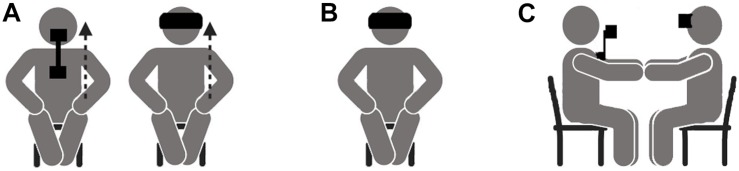
Phases of the TMTBA-VR condition. **(A)** Phase 1: Embodied illusion induction; **(B)** Phase 2: Self-compassion training; and **(C)** Phase 3: Body swap experience– Self-compassion facing oneself.

For clarity, the TMTBA-VR condition in this study was divided into three phases (Figure 1). The first phase has the purpose of generating a body swap illusion, allowing the participant to take over the body of another person (the performer) (Figure 1A). To do so, an embodied induction is performed. In this phase, the participant and the performer are sitting aligned. The participant is wearing the head-mounted display (VR Oculus Rift), which allows him/her to see the torso, legs, and arms of the performer’s body. The performer is wearing a camera controlled by the participant’s head movements. A pre-recorded instruction to perform specific movements is played to each participant (e.g., “Put your right hand on your right knee, and then slowly move it up to your lap, as if you were caressing it”). All the movements selected followed two principles: (1) movements that require a combination of visual and haptic senses, in order to increase the embodied illusion; and (2) movements that ensure the synchronization between the participant’s and performer’s movements. This phase lasts 5 min. The second phase consists of the compassion meditation itself (Figure 1B). The participant is still wearing the VR Oculus Rift, but it is turned off. A self-compassion meditation is played to the participant for 15 min. At the end of the meditation, the third phase begins (Figure 1C). The participant faces him/herself while listening to self-compassionate messages. To do so, the performer sits facing the participant. After this, the VR Oculus Rift is turned on, allowing the participant to see him/herself from a third-person perspective. Participants are invited to hug themselves. The performer follows the participant’s movements like a mirror. This phase lasts from 5 to 7 min.

**FIGURE 2:**
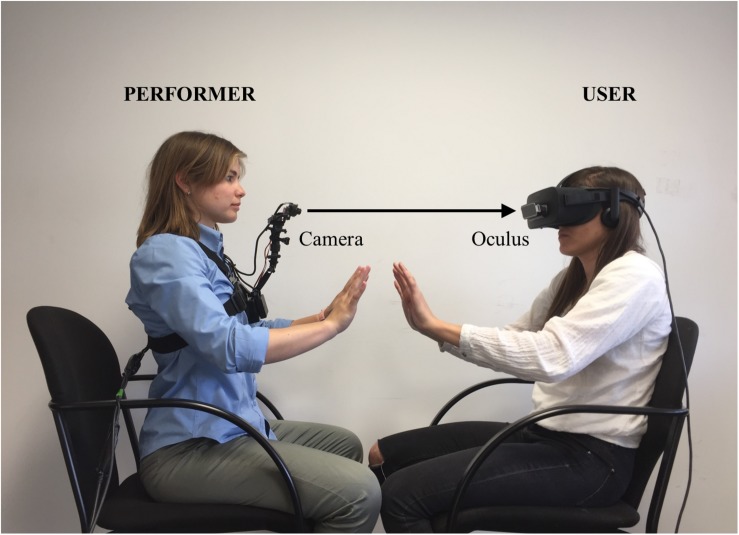
Pictures took during the experimental session of The Machine to Be Another.

**FIGURE 3:**
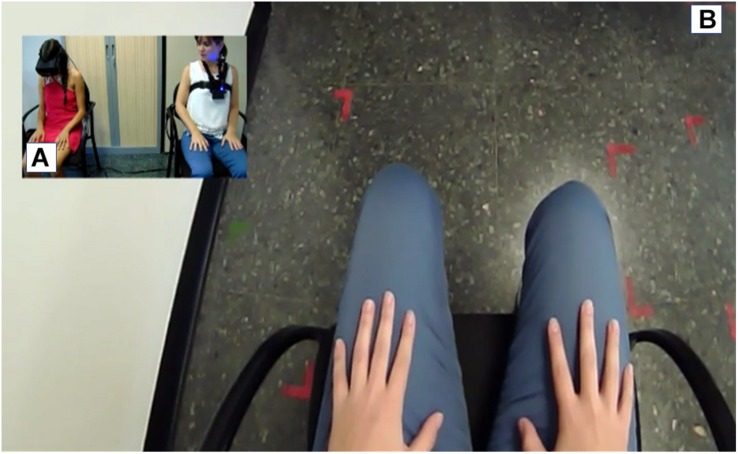
The Machine to Be Another system. **(A)** shows the synchronic movements between the performer and the user. **(B)** shows the image that user sees with the Oculus.

In order to induce this experience, the TMTBA-VR has the support of a performer, a person who is trained to mimic the user’s movements to induce the embodied illusion. Hence, in order to control the user’s movements, an audio recording is played where precise and slow movements are requested (e.g., “move your hand from your lap to your knee”). The TMTBA-VR is connected to the head-mounted display (Oculus Rift), and the performer’s first-person perspective is captured by a camera controlled by the user’s head movements, revealing the torso, legs, and arms of the performer’s body (Figure 2). Through the Oculus, the user sees the image captured by the camera, creating the illusion of being another person (Figure 3).

### Data Analyses

All statistical analyses were performed using the SPSS v.24. First, descriptive statistics, independent-samples *t*-tests, and chi-square tests were conducted to test whether there were significant differences between conditions on sociodemographic, psychological, and practice-related meditation variables, as well as on baseline measures (PHQ-9 and GAD-7). Second, mixed 2 × 2 ANOVAS, with condition (TMTBA-VR and CAU) as between-subjects factor and time (T1 and T4) as within-subjects factor, were performed to analyze the effects of the condition on SOFI and SMS, before and after the meditation practice. Third, one-sample *t*-tests were conducted to explore the effect of the TMTBA-VR on the embodiment scores (location, ownership, and agency). Fourth, an independent-samples *t*-test was performed to analyze the adherence to the meditation practice. In addition, mixed 2 × 2 ANOVAS were conducted, with condition (TMTBA-VR and CAU) as between-subjects factor and time (T2 and T3) as within-subjects factor, in order to analyze the effects of the condition on adherence, MSCS, and PANAS at baseline and after 2 weeks of meditation practice. Fifth, moderation analyses were carried out to test whether the imagery ability subscales (QMI) moderated the effect of the condition on adherence to meditation practice after 2 weeks. These analyses were performed using the procedure described by Hayes (2018) from the macro PROCESS (version 3.2), choosing model 1. In these analyses, the TMTBA-VR condition was coded as “1,” and the CAU condition as “2.” All the regression coefficients were reported in unstandardized form as *b*-values. Tests of significance (*p* < 0.05) or a confidence interval (not including zero) in the interaction between condition and the QMI subscales were carried out to find out whether the QMI moderated the effect of condition on adherence. The conditional effects of condition on adherence at medium (the mean), low (+1 SD), and high (−1 SD) levels of the QMI subscales were examined using the “pick-a-point” approach (or analysis of simple slopes).

## Results

### Differences in Sociodemographic, Psychological, and Practice-Related Meditation Variables and Baseline Measures (PHQ-9 and GAD-7) Between Conditions

Descriptive statistics for sociodemographic, psychological, and practice-related meditation variables, as well as the PHQ-9 and GAD-7 questionnaires for each condition, are shown in Table 1. There were no significant differences between conditions for age, *t*(14) = −0.89, *p* = 0.387; gender, χ^2^(1, *N* = 16) = 1.33, *p* = 0.248; history of mental or chronic illness, χ^2^(1, *N* = 16) = 0.00, *p* = 1.00; experience with meditation, χ^2^(1, *N* = 16) = 1.07, *p* = 0.302; frequency of meditation, χ^2^(1, *N* = 16) = 1.07, *p* = 0.302; PHQ-9, *t*(14) = −0.82, *p* = 0.427; or GAD-7, *t*(14) = −0.18, *p* = 0.859.

**TABLE 1:** Descriptive statistics of sociodemographic and practice-related meditation variables, and baseline measures (PHQ-9 and GAD-7) in TMTBA-VR and CAU condition.

	**TMTBA-VR** **condition(*n* = 8) *M (SD)***	**CAU condition (*n* = 8) *M (SD)***
**Sociodemographic and practice-related meditation variables**		
Age (years)	28.13 (7.18)	33.00 (13.69)
Gender (% women)	62.5%	87.5%
Education attained (%)		
Ph.D.	12.5%	25%
Master	75%	50%
Degree	0%	25%
Secondary studies	12.5%	0%
History of mental or chronic illness (% yes)	12.5%	12.5%
Experience in meditation (% yes)	75.0%	50.0%
Frequency of meditation (%)		
Never	50.0%	25.0%
Almost never	50.0%	75.0%
PHQ-9	3.13 (1.36)	4.13 (3.18)
GAD-7	10.25 (2.87)	10.50 (2.67)

*PHQ-9, patient health questionnaire-9; GAD-7, the generalized anxiety disorder questionnaire.*

### Effects of the TMTBA-VR (vs. CAU) Condition on SOFI and SMS Before (T2) and After (T3) the Compassion Practice

Descriptive statistics for SOFI and SMS at T2 and T3 are shown in Table 2. For the SOFI subscales, there were main effects of time for positive qualities toward self, *F*(1,14) = 21.30 *p* < 0.001, η^2^_p_ = 0.60; positive qualities toward others, *F*(1,14) = 9.41, *p* = 0.008, η^2^_p_ = 0.40; and negative qualities toward self, *F*(1,14) = 5.40, *p* = 0.036, η^2^_p_ = 0.28, with higher scores at T3 than at T2 in the case of positive qualities, and lower scores at T3 than at T2 in the case of negative qualities. However, the main effect of time for negative qualities toward others was not statistically significant, *F*(1,14) = 1.68, *p* = 0.216, *η^2^_p_* = 0.11. No significant interaction effects were found for any of the SOFI subscales: positive qualities toward self, *F*(1,14) = 0.66 *p* = 0.429, η^2^_p_ = 0.05; positive qualities toward others, *F*(1,14) = 0.03, *p* = 0.859, η^2^_p_ = 0.00; negative qualities toward self, *F*(1,14) = 1.57, *p* = 0.231, η^2^_p_ = 0.10; or negative qualities toward others, *F*(1,14) = 0.75, *p* = 0.402, η^2^_p_ = 0.05.

**TABLE 2:** Descriptive statistics of SOFI and SMS before (T2) and after (T3) the compassion practice, and adherence, MSCS, and PANAS at baseline (T1) and after 1 week of meditation practice (T4).

	**TMTBA-VR condition** **(*n* = 8)**			**CAU condition** **(*n* = 8)**		
	**Pre** ***M(SD)***	**Post** ***M(SD)***	**Within-group effect size *d* (95% CI)**	**Pre** ***M(SD)***	**Post** ***M(SD)***	**Within-group effect size *d* (95% CI)**
**Changes T2**−**T3**						
**SOFI**						
Positive qualities toward self	13.75 (2.71)	16.25 (1.98)	−0.82 (−1.59, −0.05)	14.25 (3.20)	16.00 (3.25)	−0.49 (−0.99, 0.02)
Positive qualities toward others	14.38 (2.72)	15.50 (2.67)	−0.37 (−0.80, 0.07)	15.88 (1.96)	16.88 (2.47)	−0.45 (−0.98, 0.07)
Negative qualities toward self	4.63 (1.06)	4.25 (0.71)	0.32 (−0.06, 0.70)	6.25 (2.55)	5.00 (2.83)	0.44 (−0.15, 1.02)
Negative qualities toward others	4.63 (1.06)	4.50 (1.07)	0.11 (−0.33, 0.55)	5.00 (1.41)	4.38 (1.06)	0.39 (−0.52, 1.30)
**SMS**						
Mental events	50.88 (11.95)	59.00 (9.80)	−0.60 (−1.46, 0.25)	47.13 (13.14)	64.63 (7.95)	−1.18 (−2.07, −0.29)
Bodily sensations	17.25 (5.55)	22.50 (4.78)	−0.84 (−2.03, 0.35)	17.75 (5.39)	24.25 (3.92)	−1.08 (−2.03, −0.11)
**Changes T1**−**T4**						
**Adherence**	–	4.25 (0.71)	–	–	3.50 (1.31)	–
**MSCS**						
Physical Care	21.25 (4.20)	22.88 (5.17)	−0.34 (−0.82, 0.13)	23.38 (4.69)	24.38 (3.58)	−0.19 (−0.80, 0.42)
Supportive relationships	21.50 (3.16)	21.75 (2.55)	−0.07 (−0.56, 0.42)	18.50 (2.73)	19.50 (1.51)	−0.33 (−1.45, 0.79)
Mindful Awareness	14.63 (3.85)	15.75 (3.15)	−0.26 (−0.89, 0.37)	12.75 (2.55)	14.25 (3.58)	−0.52 (−1.21, 0.16)
Self-compassion and purpose	18.00 (4.78)	22.13 (5.89)	−0.77 (−1.54, 0.00)	19.13 (4.52)	20.75 (4.50)	−0.32 (−1.02, 0.38)
Mindful Relaxation	15.38 (2.67)	19.00 (4.96)	−1.21 (−2.49, 0.08)	17.00 (3.02)	19.88 (4.49)	−0.85 (−1.83, 0.13)
Supportive structure	16.00 (3.34)	17.50 (1.60)	−0.40 (−1.32, 0.52)	13.25 (2.92)	14.50 (1.51)	−0.38 (−1.34, 0.58)
Clinical	16.38 (2.45)	23.00 (3.96)	−2.40 (−4.01, −0.79)	16.75 (2.92)	19.88 (3.04)	−0.95 (−2.01, 0.10)
General	6.75 (3.49)	9.75 (3.65)	−0.76 (−1.54, 0.01)	7.50 (3.07)	8.88 (1.81)	−0.40 (−1.21, 0.41)
**PANAS**						
Positive	31.75 (7.59)	32.50 (6.05)	−0.09 (−0.78, 0.61)	34.50 (7.54)	37.25 (9.00)	−0.32 (−1.09, 0.44)
Negative	18.00 (5.18)	18.00 (4.14)	0.00 (−0.86, 0.86)	19.25 (6.23)	19.50 (6.37)	−0.04 (−0.60, 0.52)

*SOFI, self-other four immeasurable scale; SMS, mindfulness state scale; MSCS, mindfulness self-care; PANAS, positive and negative affect schedule.*

Regarding the SMS subscales, there were main effects of time on mental events, *F*(1,14) = 25.66, *p* < 0.001, η^2^_p_ = 0.65, and bodily sensations, *F*(1,14) = 14.44, *p* = 0.002, η^2^_p_ = 0.51. However, there were no interaction effects between condition and time on mental events, *F*(1,14) = 3.44, *p* = 0.085, η^2^_p_ = 0.20, or bodily sensations, *F*(1,14) = 0.16, *p* = 0.692, η^2^_p_ = 0.01.

### Effect of the TMTBA-VR on Embodiment Scores

Regarding the effects of the TMTBA-VR on embodiment scores for location, *M* = 5.42, *SD* = 1.86, *t*(7) = 6.72, *p* = 0.001, ownership, *M* = 4.53, *SD* = 2.20, *t*(7) = 4.53, *p* = 0.003, and agency of the performer’s body, *M* = 4.69, *SD* = 1.94, *t*(7) = 5.36, *p* = 0.001, they were significantly greater than the score of 1 -which is equivalent to the non-experience of location, ownership, or agency of the performer’s body–.

### Effects of the TMTBA-VR (vs. CAU) Condition on Adherence, MSCS, and PANAS at Baseline (T1) and After Two Weeks of Meditation Practice (T4)

Descriptive statistics for adherence to meditation practice, MSCS, and PANAS are shown in Table 2. Regarding the effects of the condition on adherence, there were no significant differences in the days that participants had practiced meditation in the past 2 weeks, *t*(10.76) = 1.43, *p* = 0.182, *d* = 0.67, 95% CI (−0.33, 1.68).

Regarding the MSCS subscales, main effects of time were found for Self-compassion and Purpose, *F*(1,14) = 7.10, *p* = 0.018, η^2^_p_ = 0.34, Mindful Relaxation, *F*(1,14) = 6.63, *p* = 0.022, η^2^_p_ = 0.32, Clinical practices, *F*(1,14) = 39.51, *p* < 0.001, η^2^_p_ = 0.74, and General practices, *F*(1,14) = 9.25, *p* = 0.009, η^2^_p_ = 0.40, with higher scores at T4 than in T1. However, there were no main effects of time for Physical Care, *F*(1,14) = 2.65, *p* = 0.126, η^2^_p_ = 0.16, Supportive Relationships, *F*(1,14) = 0.85, *p* = 0.373, η^2^_p_ = 0.06, Mindful Awareness, *F*(1,14) = 3.42, *p* = 0.086, η^2^_p_ = 0.20, or Supportive structure, *F*(1,14) = 3.04, *p* = 0.103, η^2^_p_ = 0.18. Significant interaction effects between condition and time were only found for Clinical practices, *F*(1,14) = 5.09, *p* = 0.041, η^2^_p_ = 0.27. *Post hoc* analysis using Bonferroni correction showed that both conditions increased from T1 to T4 (TMTBA-VR: *p* < 0.001; CAU: *p* = 0.013), and a trend toward statistically significant differences was found between conditions at T4 (*p* = 0.099), with higher scores on the TMTBA-VR than in the CAU condition. By contrast, no interaction effects were found for the rest of the subscales: Self-compassion and Purpose, *F*(1,14) = 1.34, *p* = 0.266, η^2^_p_ = 0.09, Mindful Relaxation, *F*(1,14) = 0.09, *p* = 0.771, η^2^_p_ = 0.01, General, *F*(1,14) = 1.28, *p* = 0.278, η^2^_p_ = 0.08, Physical Care, *F*(1,14) = 0.15, *p* = 0.704, η^2^_p_ = 0.01, Supportive Relationships, *F*(1,14) = 0.30, *p* = 0.590, η^2^_p_ = 0.02, Mindful Awareness, *F*(1,14) = 0.07, *p* = 0.796, η^2^_p_ = 0.01, and Supportive structure, *F*(1,14) = 0.03, *p* = 0.876, η^2^_p_ = 0.00.

Regarding PANAS, there were no main effects of time on positive PANAS, *F*(1,14) = 0.86, *p* = 0.370, η^2^_p_ = 0.02, or negative PANAS, *F*(1,14) = 0.01, *p* = 0.925, η^2^_p_ = 0.00. Moreover, there were no significant interaction effects between condition and time on positive PANAS, *F*(1,14) = 0.28, *p* = 0.605, η^2^_p_ = 0.02, or negative PANAS, *F*(1,14) = 0.01, *p* = 0.925, η^2^_p_ = 0.00.

### Imagery Ability (QMI) as a Moderator of the Effect of Condition on Adherence to Meditation Practice After Two Weeks

**FIGURE 4:**
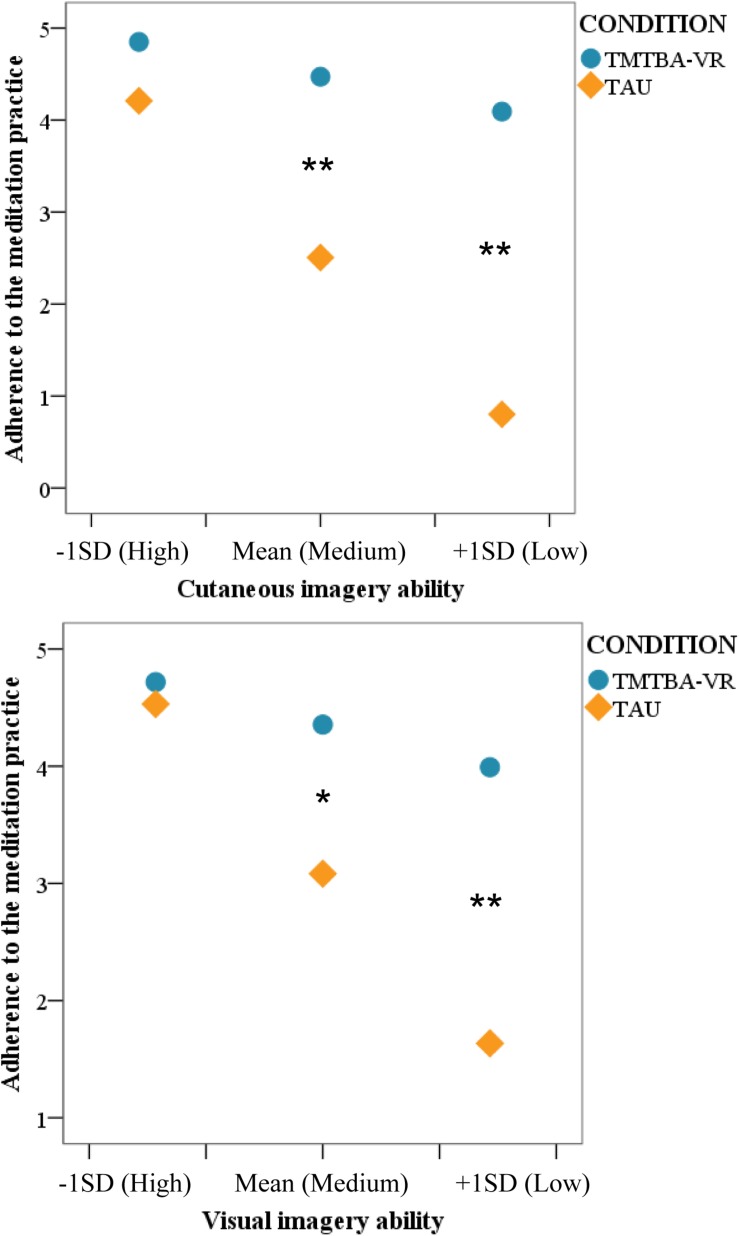
Simple slopes graph of the regressions of condition on meditation practice after 2 weeks at three levels of the moderator variables (low, moderate, and high). 1 = never; 2 = 1–2 times; 3 = 3–4 times; 4 = 5–6 times; 5 = Every day. “Low,” “medium,” and “high” levels of the moderators represent the mean and ± 1 standard deviation (SD): Visual imagery ability (range: 4–28) = 12.16 (M) ± 7.97 (SD); Cutaneous imagery ability (range: 5–35) = 16.35 (M) ± 8.67 (SD). Significant *p*-value (^*^p < 0.05, ^∗∗^*p* < 0.01) represent the level of the moderator in which the conditional effect of condition on meditation practice is significant.

Moderation analyses showed that the specific QMI subscales moderated the effect of condition on adherence: cutaneous, *F*(1,12) = 5.95, *p* = 0.031, and a trends of statistical significance in visual, *F*(1,12) = 3.65, *p* = 0.080 (see Figure 4). However, the following imagery ability factors did not moderate this relationship: gustatory and olfactory, *F*(1,12) = 0.56, *p* = 0.468; kinesthetic, *F*(1,12) = 0.03, *p* = 0.866; organic, *F*(1,12) = 0.50, *p* = 0.495; and auditory, *F*(1,12) = 0.06, *p* = 0.808.

Regarding the first significant moderation model with cutaneous imagery ability as moderator, the overall model explained 54.95% of the variance in adherence, *F*(3,12) = 6.11, *p* = 0.009. The interaction between condition and adherence was significant, *F*(1,12) = 5.95, *p* = 0.031, which means that cutaneous imagery (QMI) was a moderator of the effect of the condition on adherence, accounting for 16.86% of the variance. Simple slopes analysis showed that there was a significant negative relationship between condition and adherence when cutaneous imagery was “medium,” *b* = −1.97, 95% CI (−3.16, −0.77), *t* = −3.57, *p* = 0.004, and “low,” *b* = −3.29, 95% CI (−5.28, −1.30), *t* = −3.61, *p* = 0.004. Participants in the TMTBA-VR (vs. CAU) had greater adherence to the meditation practice when cutaneous imagery was “medium” (TMTBA-VR = 4.47 ≈ 5–6 times; CAU = 2.51 ≈ 3–4 times) and “low” (TMTBA-VR = 4.09 ≈ 5–6 times; CAU = 0.80 ≈ never).

With regard to the second relevant moderation model with visual imagery ability as moderator, the overall model explained 51.66% of the variance in adherence, *F*(3,12) = 7.50, *p* = 0.004. The interaction between condition and adherence showed a trend of statistical significance, *F*(1,12) = 3.65, *p* = 0.080, which means that visual imagery (QMI) could be a moderator of the effect of the condition on adherence, accounting for 13.98% of the variance. Simple slopes analysis showed that there was a negative significant relationship between condition and adherence when visual imagery was “medium,” *b* = −1.27, 95% CI (−2.18, −0.36), *t* = −3.05, *p* = 0.010, and “low,” *b* = −2.36, 95% CI (−3.77, −0.94), *t* = −3.63, *p* = 0.004. Participants in TMTBA-VR (vs. CAU) had greater adherence to meditation practice when visual imagery was “medium” (TMTBA-VR = 4.36 ≈ 5–6 times; CAU = 3.08 ≈ 3–4 times) and “low” (TMTBA-VR = 3.99 ≈ 5–6 times; CAU = 1.63 ≈ 1–2 times).

## Discussion

The general objective of the present study was to analyze the effects of a self-compassion meditation supported by an embodied VR system (TMTBA-VR), compared to usual practice (only audio).

Regarding the results before and after the meditation, it was expected an increase in positive qualities toward self/others and a decrease in negative qualities toward self/others, as a measure of the compassion effects. Both conditions increased the positive qualities toward self and others and decreased the negative qualities toward self. There were no differences between conditions across time, but an exploration of the effect sizes showed that the effect of positive qualities toward self was larger in the TMTBA-VR condition than in the CAU condition. Thus, our hypothesis that the VR system could facilitate access to the powerful emotional response of self-compassion was not fully supported. Different factors could explain this lack of evidence and need to be further study, such as the sample size or the characteristics of participants (all were university students). Even though, a promising result was found in the difference between groups, as an exploration of the effect sizes showed that the effect of positive qualities toward self was larger in the TMTBA-VR than in the CAU condition. In this sense, we would like to highlight the experience of awe as a potential explanation of this difference. Most participants in the TMTBA-VR condition reported a sense of awe generated by the experience of embodying another’s body and touching themselves, and this experience could be interacting with the self-compassion response. As was previously reported in other studies, VR is an effective way to induce awe in controlled experimental settings, due to its ability to provide participants with a sense of “presence,” that is, the subjective feeling of being displaced in another physical or imaginary place (Chirico et al., 2018). Unfortunately, we did not use a questionnaire to measure awe in order to control this experience. Furthermore, there is no previous research about the efficacy of a single meditation supported by VR in generating positive mental states. Thus, further studies are needed to verify the sense of awe triggered by the VR experience and its effects on the practice and adherence to the practice. Regarding this point, it could be controlled in future studies allowing the participants to habituate to the TMTBA previous to study its efficacy.

Both conditions increased awareness and attention to the present for mental events and bodily sensations after the compassion practice, with no differences between conditions across time. A mindfulness state refers to the mental ability to pay attention to physical or mental events that occur in the present moment (Tanay and Bernstein, 2013), which means that the TMBTA-VR experience does not distract from body sensations and the mind, an essential aspect of self-compassion and other meditations (Cebolla et al., 2017, 2018). However, explorations of the effect sizes showed that both factors were larger in the CAU condition than in the TMTBA-VR condition.

Regarding the second objective of this study, it was hypothesized that TMTBA-VR would increase adherence to meditation practice by supporting the construction of the self-compassionate image. Both conditions practiced a similar number of days after 1 week, around 4 times per week. The characteristics of the sample could explain these unexpected results and future research is needed to test this results in participants who score high on fear of compassion, self-criticism, or lack of imagination skills.

With regard to the frequency of self-care behaviors, the results showed that both conditions increased the scores on the frequency of self-care behaviors, such as self-compassion and purpose (e.g., acceptance of failure and challenge as part of the process), mindful relaxation (e.g., doing specific practices that can help individuals to relax), clinical self-care practices (e.g., resting when individuals needed to), and general self-care practices (e.g., engaging in a variety of self-care strategies). Explorations of the effect sizes showed that participants in the TMTBA-VR condition achieved larger effects than in the CAU condition, especially on the clinical self-care subscale. In fact, an interaction effect between condition and time was found for this subscale. Nevertheless, the difference between conditions at 2 weeks did not reach statistical significance, and the results derived from this subscale –as well as the mindful relaxation subscales– should be viewed with caution because the internal consistencies of these subscales were not adequate. This result is interesting because in the experiment both conditions had similar scores pre-post, which means that the home practice is where participants generate different states that contribute to different mindful self-care responses. Further research should analyze the quality of the practice at home and the ability of this practice to produce positive mental states, as well as the impact on self-care responses.

The secondary objective of this study was to analyze whether the imagery ability was a moderator of the effect of the condition on adherence to meditation practice after 2 weeks. Analyses showed that adherence was significantly different between conditions when the cutaneous and visual (only a trend) imagery abilities were introduced as moderators (although the interaction was marginally significant in the case of the visual imagery ability). Participants in the TMTBA-VR (vs. CAU) had greater adherence to the meditation practice when cutaneous and visual imagery were “medium” and “low” in this sample. Thus, when visual and cutaneous imagery were lower, participants in the TMTBA-VR condition practiced a minimum of 5–6 times per week, whereas participants in the CAU condition practiced a maximum of 3–4 times per week (and even “never” when cutaneous imagery was low). This result is consistent with the main hypothesis proposing that VR could facilitate a powerful self-compassion experience in participants, and that imagery could be used in daily practice, increasing adherence. Furthermore, this effect seems to be more powerful in people who show a lack of imagination skills, which means that VR could be especially relevant in clinical samples where imagery has been shown to play a key role (Pearson et al., 2013). This results is congruent with the importance of mental imagery in the area of clinical psychopathology (Hackmann et al., 2011) and psychological interventions (Gilbert and Irons, 2004). Embodied VR can be especially relevant when constructive meditations are used in CBIs, given the key role of imagery. Even though, it is important to highlight that not only imagery skills are important in compassion practice. Others factors like body awareness or body representations could play a key role in the practice. However, few researches has been done in this area, and further studies should be conducted in the future.

Despite the several limitations of the present pilot study pointed out in the current study (including the small sample size, the participant’s characteristics given that they are students with high level of education with no mental disorder) it is important to highlight that this study is one of the first study that proposed the use of VR as a tool to improve compassion practice. Using VR as support to the compassion training may help to overcome several difficulties that trainee experience in the early state of their training, especially those related to the imagery skills. After this study several question arise that need to be study to better understand the role of the VR in the training and its effects. In this sense more research is needed for example to test the effects of the TMTBA-VR in clinical population, the role played by imagery skill levels, awe, and other relevant variables like self-criticism. Regarding this point, it could be controlled in future studies allowing the participants to habituate to the TMTBA previous to study its efficacy.

## Ethics Statement

The study was approved by The Ethics Committee at the University of Valencia (Spain), with registration number: H1513592028862.

## Author Contributions

AC and RH made substantial contribution to the conceptualization, formal analyses, and drafting the manuscript. SV and MB-B made substantial contribution to the collection of the data. MM made substantial contribution to the forma analyses and drafting the manuscript. RB made substantial contribution in revising the manuscript critically for important intellectual content. All authors provided final approval of the version to be published, and agree to be accountable for all aspects of the work in ensuring that questions related to the accuracy or integrity of any part of the work are appropriately investigated and resolved.

## Conflict of Interest Statement

The authors declare that the research was conducted in the absence of any commercial or financial relationships that could be construed as a potential conflict of interest.

## Abbreviations

CBIs: compassion-based interventions
GAD-7: the generalized anxiety disorder questionnaire
MSCS: mindfulness self-care
PANAS: positive and negative affect schedule
PHQ-9: patient health questionnaire-9
QMI: Betts’ questionnaire upon mental imagery
SMS: state mindfulness scale
SOFI: self-other four immeasurable scale
TMTBA: the machine to be another
VR: virtual reality.
